# An Eighteenth-Century Hospital

**Published:** 1921-09-24

**Authors:** 


### An Eighteenth-Century Hospital.

A number of interesting facts about the Leicester Royal Infirmary was given in a recent address at the Leicester Rotary Club by Mr. Harry Johnson on the "Work of the Royal Infirmary."

The institution owed its being to one Dr. Watts, of Medbourne, one-time physician to the Northampton Infirmary, who, in the year 1766, submitted a touching appeal in the characteristic language of that day urging the residents of Leicestershire to follow other county towns, such as Winchester, Northampton, and Newcastle, and found a hospital. At that time the population of Leicester was about 10,000, and except for the workhouse no hospital existed. The funds having been raised, eventually the Chappell Close site was secured. As its name implies, this Close at one time was the burying-ground attached to St. Sepulchre's Church. The church faced the public gallows. The bodies of malefactors who were hung were afterwards taken down to the cemetery of St. Sepulchre's for burial. This probably accounts for many bones which were found in digging the new foundations of the Infirmary on various occasions. Henry of Knighton relates the story of a man who had been hung and revived on the way to burial in this cemetery. Edward III., who happened to be staying at Leicester Abbey at the time, heard of the occurrence, and sent the man a free pardon. Some years after another man, after hanging, revived as he lay before the high altar of St. Sepulchre. He was promptly taken back to the gallows and hanged over again.

Plans and estimates for a substantial building of brick were invited at an approximate cost of £1,500. The successful tender was accepted at £2,200. The Infirmary was opened in 1771 by the then Bishop of Lincoln. The main walls of the building are of 18-in. brickwork, and still form part of the present foundations. The building was originally intended for forty beds, but by means of using service rooms and the Committee Room as wards accommodation was found for sixty patients.

In 1781 the need for establishing a lunatic asylum became acute, as there was no available accommodation for lunatics. The lunatic asylum formed part of the buildings of the Infirmary for something like sixty years, until the care of lunatics passed to the county authorities under Act of George IV. The funds of the asylum, accumulated from legacies and special gifts, were transferred with the asylum, and now represent part of a Charity Fund enabling deserving local residents to be treated as private or paying patients.

In 1819 a fever hospital was provided. Here again the Infirmary was a local pioneer, and until within present memory the fever hospital continued its existence at the Infirmary.

Exactly one hundred years ago applications for admission were so numerous that the surgeons were granted the power of admitting patients to the Committee Room. The condition of the institution at this time is described in graphic language in the minutes: "The Committee have to defer their report as to the state of the buildings in consequence of the delay which has occurred in erecting the water closets from the long confinement of a patient with a mortified leg, which made it difficult to give any opinion as to the state of the ventilation itself."

In the year 1833 the inadequacy of the Infirmary, the fever house, and the lunatic asylum reached a climax. A Committee was thereupon appointed, of which Sir Henry Halford, successively physician to George III., George IV., and William IV., was a member. This Committee, having reported the inadequacy of the then asylum (which was on a level with and close to a common sewer), was authorised to treat under the Act referred to with the magistrates of the county. Then the Committee acquired the asylum buildings for the General Infirmary. A bad bargain was made at £750, for a few months later the buildings proved quite unsuitable, and were subsequently demolished.

Flashlights on the Past.

The first matron was a widow, whose salary was fixed at £10 per annum, with a gratuity of £5 "if she behaves herself."

An apothecary from Dorset was the first resident apothecary. He was engaged at £25 per annum, "with a like gratuity to the matron."

The porter received £6 per annum, with a gratuity of £1; labourer, £5, without a gratuity; the cook £6; the laundry maid £5.

Widow Throsby and Mrs. Simonds were appointed as nurses at £6 per annum.

The standing of the nurses is graphically described: "Order'd that John Whatton, Nurse in Bottom (ward) take the place of Nurse Bonner." A little later: "Order'd that the dispensary boy be discharged and John Whatton succeed him."

Such persons as were able were employed in nursing the other patients, in washing and ironing the linen, cleaning the wards, and doing other services the matron requires—"*the apothecary being first asked if such employment is likely to interfere* with the medicines prescribed."

The matron was "order'd to buy wheat, have it ground, and made into bread at home, and provide the bags and other necessary articles for the purpose."

Thrift was enjoined on all in a manner to be commended to present-day departments: "The Committee having reason to believe that more coals are consumed than are needful, the apothecary is directed to attend to all the fires, and see that the nurses burn no more coals than are absolutely necessary; also to see that the chimnies in the nurses' room are pulled down and the grates rendered useless." No indefinite economy about this!

The Committee in those early days realised the value of newspaper advertisements when they "Order'd four persons cured to be inserted in Gregory's paper," in which an advertisement was also to be inserted "that for the future all people recommending patients must deposit a guinea instead of twelve shillings, that sum not being found sufficient for the burying of those that dye in the house."

Time was not of importance, for we read, "Discharg'd this day cured, Jonathan Bennett, after having been forty-four weeks in the house, and cur'd or reliev'd by taking off one of his toes."

